# Persistence of Long-lived Memory B Cells specific to Duffy Binding Protein in individuals exposed to *Plasmodium vivax*

**DOI:** 10.1038/s41598-018-26677-x

**Published:** 2018-05-29

**Authors:** Siriruk Changrob, Amy M. McHenry, Myat Htut Nyunt, Jetsumon Sattabongkot, Eun-Taek Han, John H. Adams, Patchanee Chootong

**Affiliations:** 1 Department of Clinical Microbiology and Applied Technology, Faculty of Medical Technology, Mahidol University, Bangkok, 10700 Thailand; 2 Department of Biological Sciences, Southwestern Adventist University, Keene, Texas 76059 USA; 3 Department of Medical Environmental Biology and Tropical Medicine, School of Medicine, Kangwon National University, Chuncheon, Gangwon-do 200-701 Republic of Korea; 4 Mahidol Vivax Research Unit, Faculty of Tropical Medicine, Mahidol University, Bangkok, 10400 Thailand; 5 Department of Global Health, University of South Florida, Tampa, Florida 33612 USA

## Abstract

The major challenge in designing a protective Duffy binding protein region II (DBPII)-based vaccine against blood-stage vivax malaria is the high number of polymorphisms in critical residues targeted by binding-inhibitory antibodies. Here, longevity of antibody and memory B cell response (MBCs) to DBL-TH variants, DBL-TH2, -TH4, -TH5, -TH6 and -TH9 were analyzed in *P. vivax*-exposed individuals living in a low malaria transmission area of southern Thailand. Antibody to DBL-TH variants were significantly detected during *P. vivax* infection and it was persisted for up to 9 months post-infection. However, DBL-TH-specific MBC responses were stably maintained longer than antibody response, at least 3 years post-infection in the absence of re-infection. Phenotyping of B cell subsets showed the expansion of activated and atypical MBCs during acute and recovery phase of infection. While the persistence of DBL-TH-specific MBCs was found in individuals who had activated and atypical MBC expansion, anti-DBL-TH antibody responses was rapidly declined in plasma. The data suggested that these two MBCs were triggered by *P. vivax* infection, its expansion and stability may have impact on antibody responses. Our results provided evidence for ability of DBPII variant antigens in induction of long-lasting MBCs among individuals who were living in low malaria endemicity.

## Introduction

*Plasmodium vivax* is being increasingly recognized as more than 16 million cases occur each year and more than a third of the world’s population is at risk of *P. vivax* infection^1,2^. Vaccine-induced protective immunity boosted by natural exposure to *P. vivax* might extend the duration of vaccine protection and therefore assist in control and eradication of *P. vivax* malaria infection^3^. The interruption of merozoite invasion into RBCs by targeting a critical ligand–receptor interaction between the *P. vivax* Duffy-binding protein region II (DBPII) and Duffy antigen receptor for chemokine (DARC) on the surface of human RBCs provides an important approach to developing a vaccine against *P. vivax*^4,5^.

Duffy binding protein (DBP) is a promising vaccine candidate against *P. vivax* malaria because naturally infected individuals produce antibodies to DBP region II (DBPII) and these naturally-acquired antibodies are capable of blocking binding of DBPII to RBCs and *P. vivax* grown *in vitro*, as are vaccine-induced anti-DBPII antibodies^6–9^. Given the importance of the DBPII antigen, the presence of naturally-acquired DBPII-specific high-level binding-inhibitory antibodies (BIAbs) in PNG children is associated with anti-malarial protection by reducing risk of high-density parasitemia^7^. The major challenge of DBPII-based vaccine development is the highly polymorphic nature of this molecule. The polymorphic residues of DBPII occur primarily in dominant B cell epitopes that are the targets of neutralizing antibodies^10^. Two strategies have been developed for *P. vivax* vaccine development to focus immune responses to conserved neutralizing DBPII epitopes: (1) synthesis of a single-allele antigen lacking polymorphic sites, (2) synthesis of a multi-allele antigen encompassing major haplotypes in malaria endemic areas. A study comparing these two DBP vaccine strategies found that both the DEKnull-based vaccine, with the removal of the dominant variant B-cell epitope, and the mixed-allele vaccine induce strain-neutralizing antibody responses but mixed-allele strategies showed more potential for generating inhibitory antibodies against a broader range of *dbpII* alleles^11,12^. Further optimization will be required to enhance efficacy.

Although there is a general consensus that antibodies are crucial for protective immunity, development and persistence of memory B cells (MBCs) following malaria infection is poorly understood. It is widely believed that long-lived anti-malarial antibodies and development of MBCs and long–lived plasma cells could be maintained by constant antigenic stimulation^13,14^ and the dormant hypnozoite of *P. vivax* could provide this stimulation. However, there is hypothesis proposes that MBCs may survive without antigen stimulation to maintain circulating antibodies, as MBCs are still identified when serum antibodies no longer existed^15^. One factor that may affect the longevity of antibodies and MBCs is parasite intensity in malaria endemic areas. In low transmission areas, antibody and MBC responses to human malaria parasites were stably maintained over 6 years without reinfection^16^. Contrastly, antibody seropositivity and circulating MBCs were not detected in children living in high transmission areas^17^. These finding reflect the potential role of transmission intensity in long-lived MBC responses.

Previous reports have shown that the antibody response to DBPII is boosted in natural infection as anti-DBPII titers increase according to the age^18,19^. However, the ability of this antigen to induce the immune system to develop and maintain antibody and MBC responses is poorly understood. An initial observation in Thai adults living in low malaria transmission areas showed stability of seropositivity to DBP antigen for up to 12 months^16^. Similarly, a cohort study in rural Amazonians showed maintenance of their inhibitory activity up to 37 months in the absence of repeated exposure to the parasite. This persistence of antibody response confers protection from clinical vivax malaria^20^. A recent study demonstrated that strain-transcending antibodies against the DBPII antigen can be safely induced by human vaccination^21^. These vaccine-induced antibodies inhibited the binding of homologous and heterologous variants of DBPII to the DARC receptor. However, previous studies lack data supporting the development and maintenance of DBPII-specific MBC responses.

In this study, we provide supporting data for the development of a DBPII-based vaccine by studying the persistence of antibody and MBC responses to DBPII and whether DBPII polymorphisms interrupt the development and maintenance of antibody and MBCs in natural infections. The longevity of levels and inhibitory function against erythrocyte binding of antibodies as well as the presence of MBCs specific to polymorphic strains of DBPII in natural *P. vivax* exposure were demonstrated.

## Results

### Organization of study subjects

**Figure 1:**
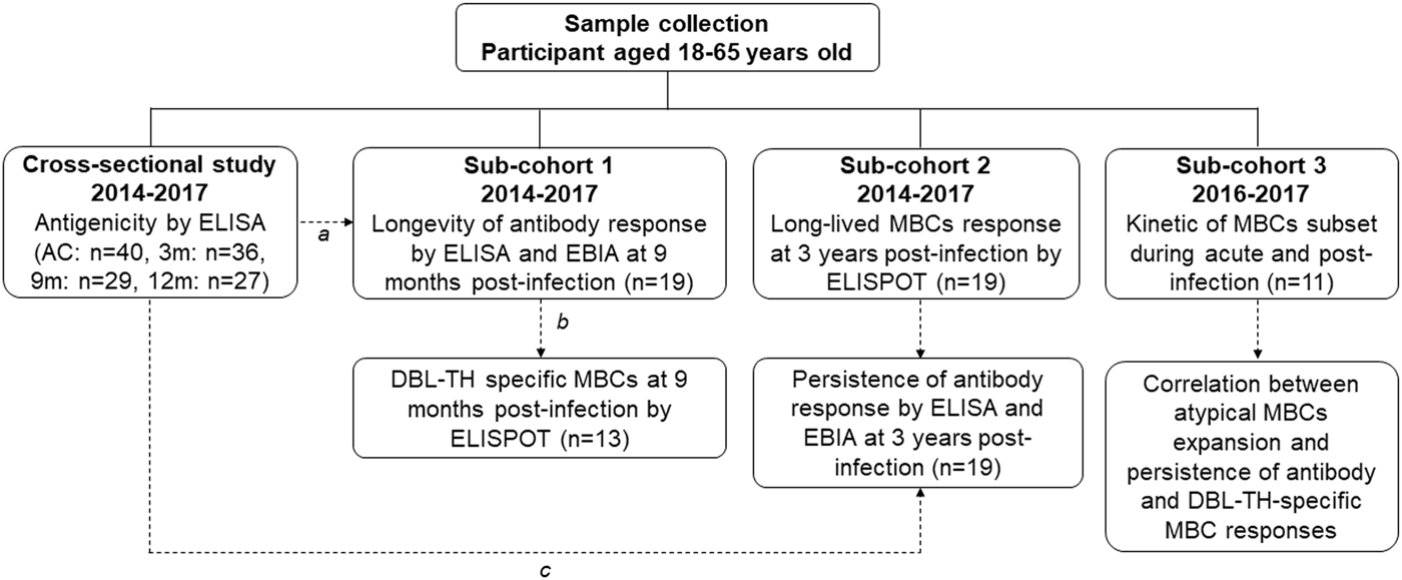
Study design. Schematic shown a flow work of three cross-sectional groups used in this study. Each dashed line refers to the sample which was taken for further experiment (**a**) subjects who had completely follow up at all time points of study, (**b**) subjects who had PBMCs available for ELISPOT assay at 9 m post-infection and (**c**) DBL-TH2 and -TH4 seropositive subjects at acute phase was follow up after recovery from infection for 3 years.

**Figure 2:**
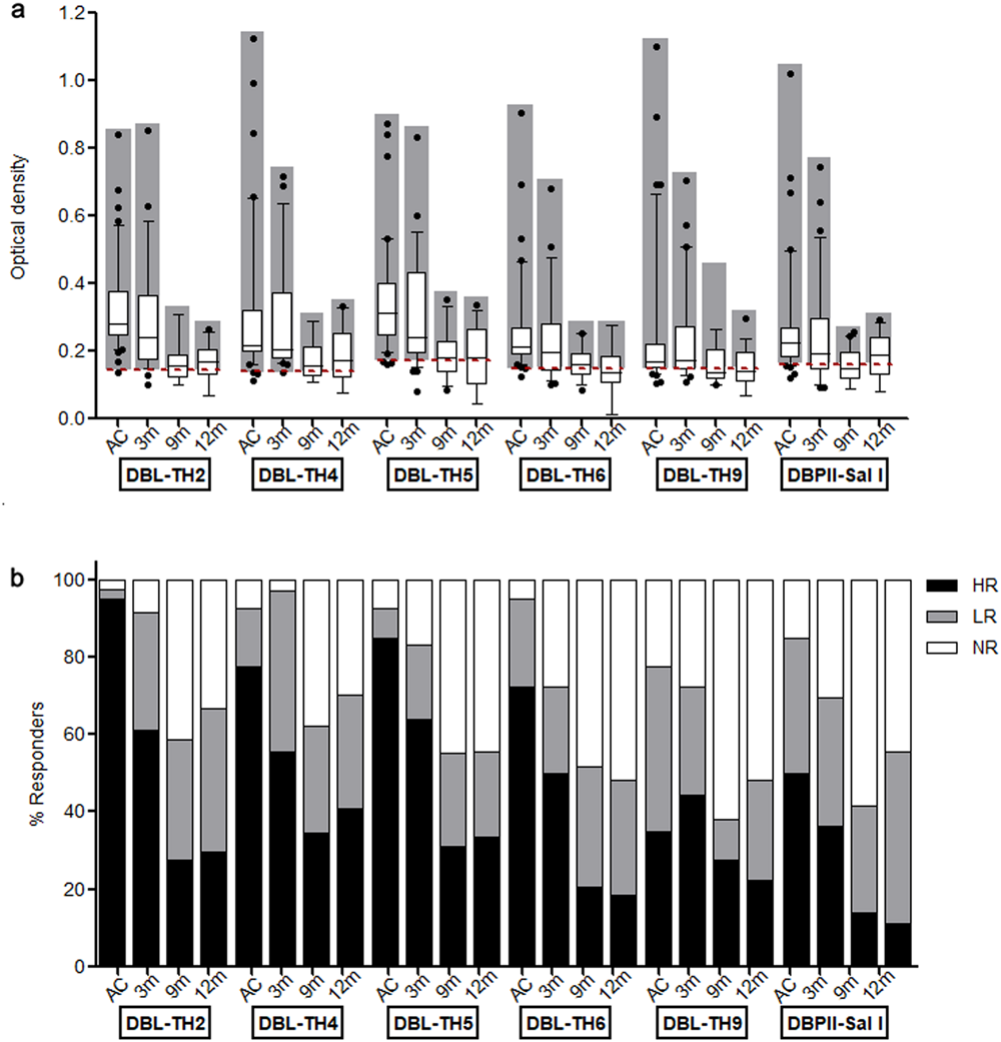
Antigenicity of polymorphic DBL-TH antigens at acute and convalescence phase of *P. vivax* infection. The total IgG antibody response against a panel of DBL-TH variant antigens and DBPII-Sal I was measured by conventional ELISA method. Human plasma from acutely infected *P. vivax* patients (AC, n = 40), 3-months post-infection (3 m, n = 36), 9 months post-infection (9 m, n = 29,) and 12-months post-infection (12 m, n = 27) were tested for antibody detection specific for DBP antigens, DBPII- Sal I, DBL-TH2, -TH4, -TH5, -TH6, and -TH9. (**a**) Box plot with 10^th^- and -90^th^-percentile values represented IgG response against antigens tested at each time point. The dashed line represents the cut-off value for each antibody response to a relative antigen equal to the mean plus 2 SD of the OD of healthy control. (**b**) The intensity of responder was classified into NR (white bar) refers to negative responders (<cut-off); LR (grey bar) refers to low responders (>cut-off but <cut-off + 2 SD of the OD of HC); HR refers to high responders (≥cut-off + 2 SD of the OD of HC).

**Figure 3:**
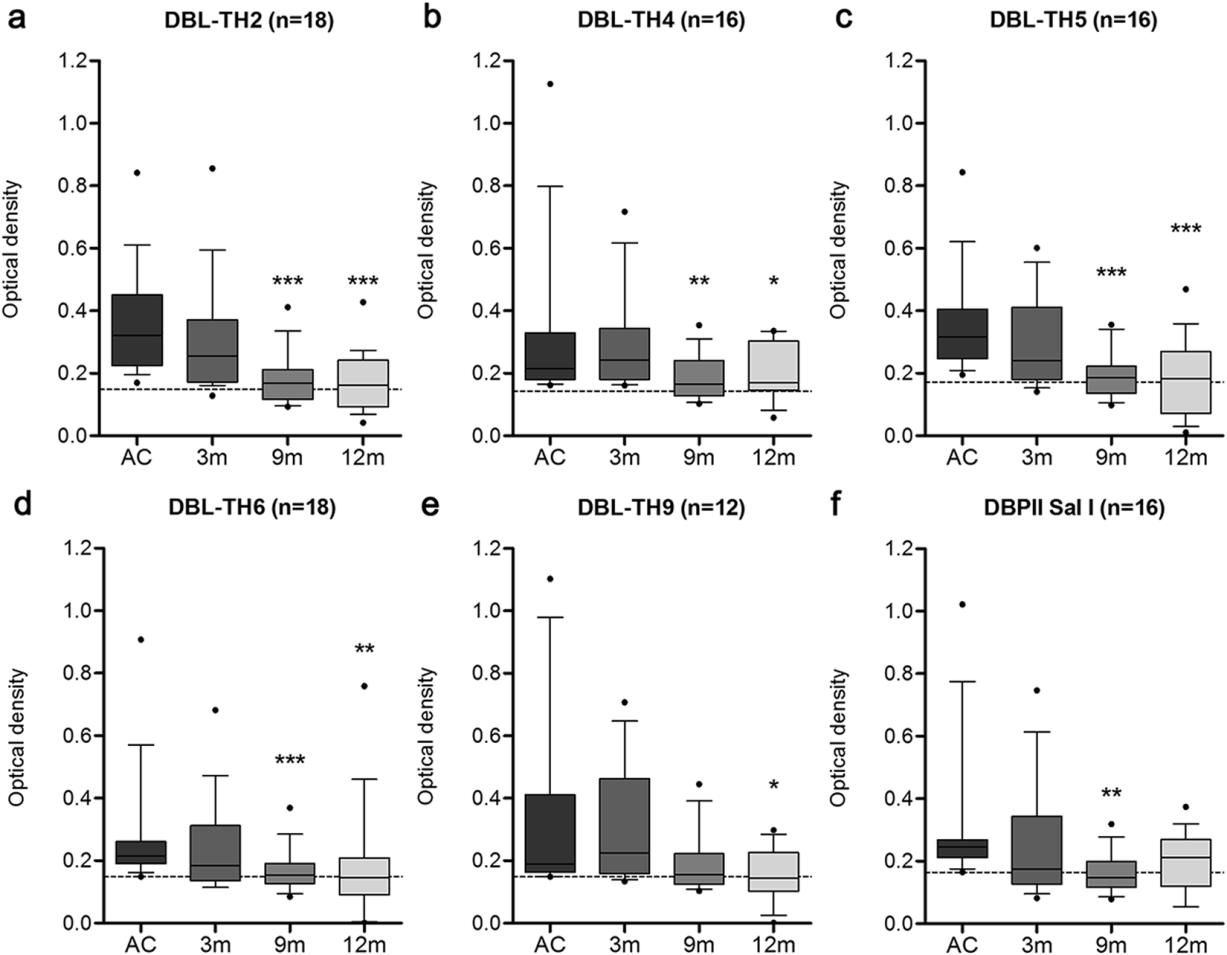
Longevity of anti-DBL-TH IgG responses. Anti-DBL-TH responses were monitored in patients during acute infection and after they recovered from infection at 3 m, 9 m and 12 m. Mean value of ELISA OD comparison of (**a**) DBL-TH2, (**b**) -TH4, (**c**) -TH5, (**d**) -TH6, (**e**) -TH9, and (**f**) DBPII-Sal I is shown as a box plot, with 10^th^- and -90^th^-percentile values represented by the bottom and top edges of the box. The dashed line represents the cut-off value for each antibody response to a relative antigen calculated as the mean plus 2 SD of the OD of HC.

**Figure 4:**
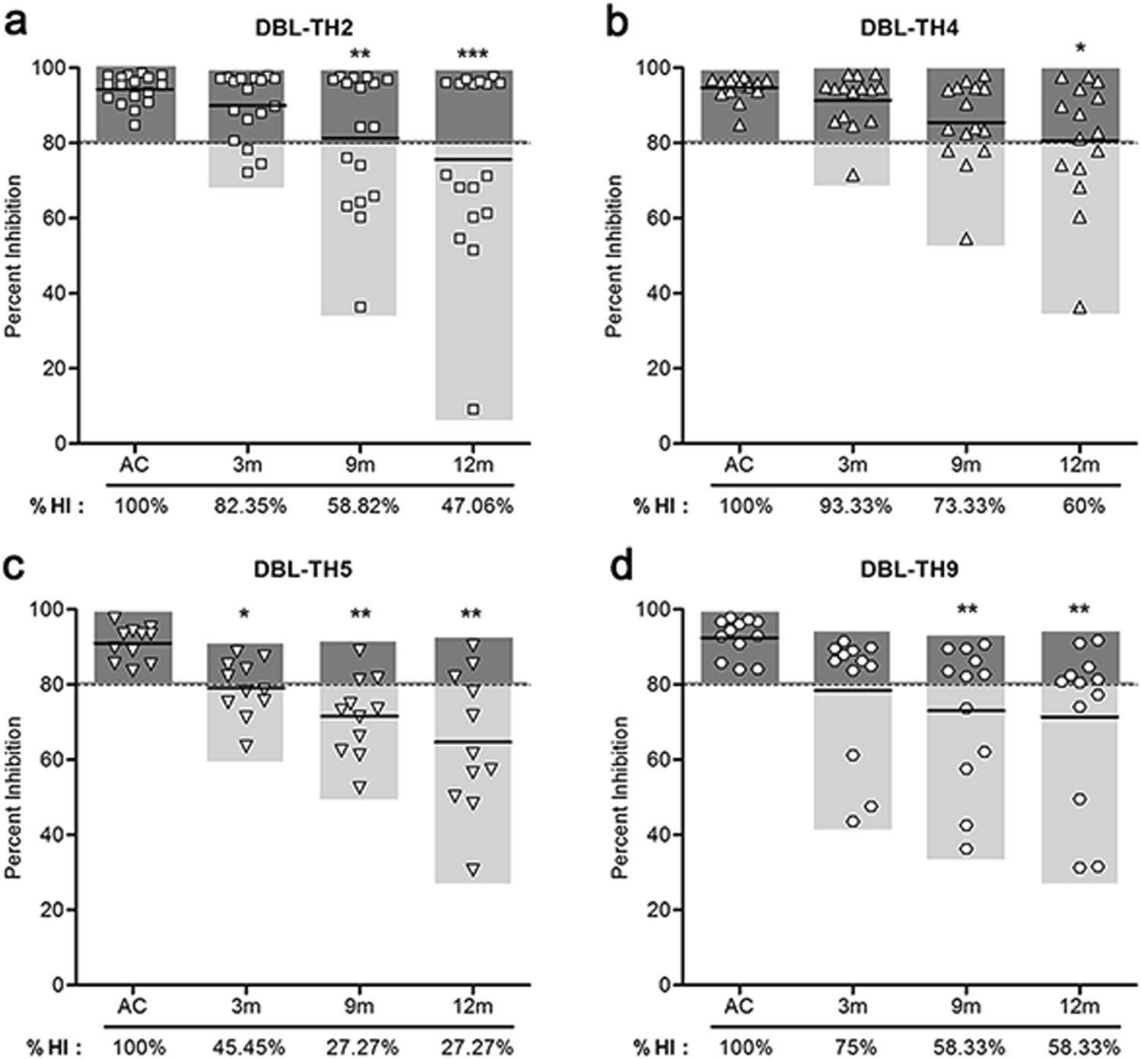
Inhibitory function of antibody against DBL-TH-erythrocyte binding was persistent post-infection. The prevalence and stability of inhibitory antibodies against (**a**) DBL-TH2, (**b**) -TH4, (**c**) -TH5 and (**d**) -TH9 binding was measured at 3 m, 9 m and 12 m post-infection. The prevalence of high inhibition was shown as %HI.

There are four distinct groups of data in this study (Fig. 1). First, cross-sectional study was recruited for surveying antigenicity of DBL-TH antigens at acute and recovery phase of *P. vivax* infection, using plasma samples collected from acutely infected *P. vivax* patients (AC, n = 40) and from subjects who recovered from infection. Each of these acute *P. vivax* patients was dropped out during survey, thus, there were 36, 29 and 27 samples collected at 3 m, 9 m and 12 m post-infection, respectively (Supplementary Table S2 and Fig. 2). Of the 40 *P. vivax*-infected individuals at enrollment, 19 individuals were completable to follow-up at all-time point of the study. Thus, 19 individual plasma samples were organized in sub-cohort 1 study and it was used for demonstrating longevity of anti-DBL-TH responses (Supplementary Table S3 and Figs 3 and 4). In addition, to correlate longevity of antibody with MBC response following *P. vivax* infection, from total 19 *P. vivax-*exposed subjects in this sub-cohort study, 13 subjects who had available PBMCs samples at 9 m post-infection were taken for ELISPOT assay.

**Figure 5:**
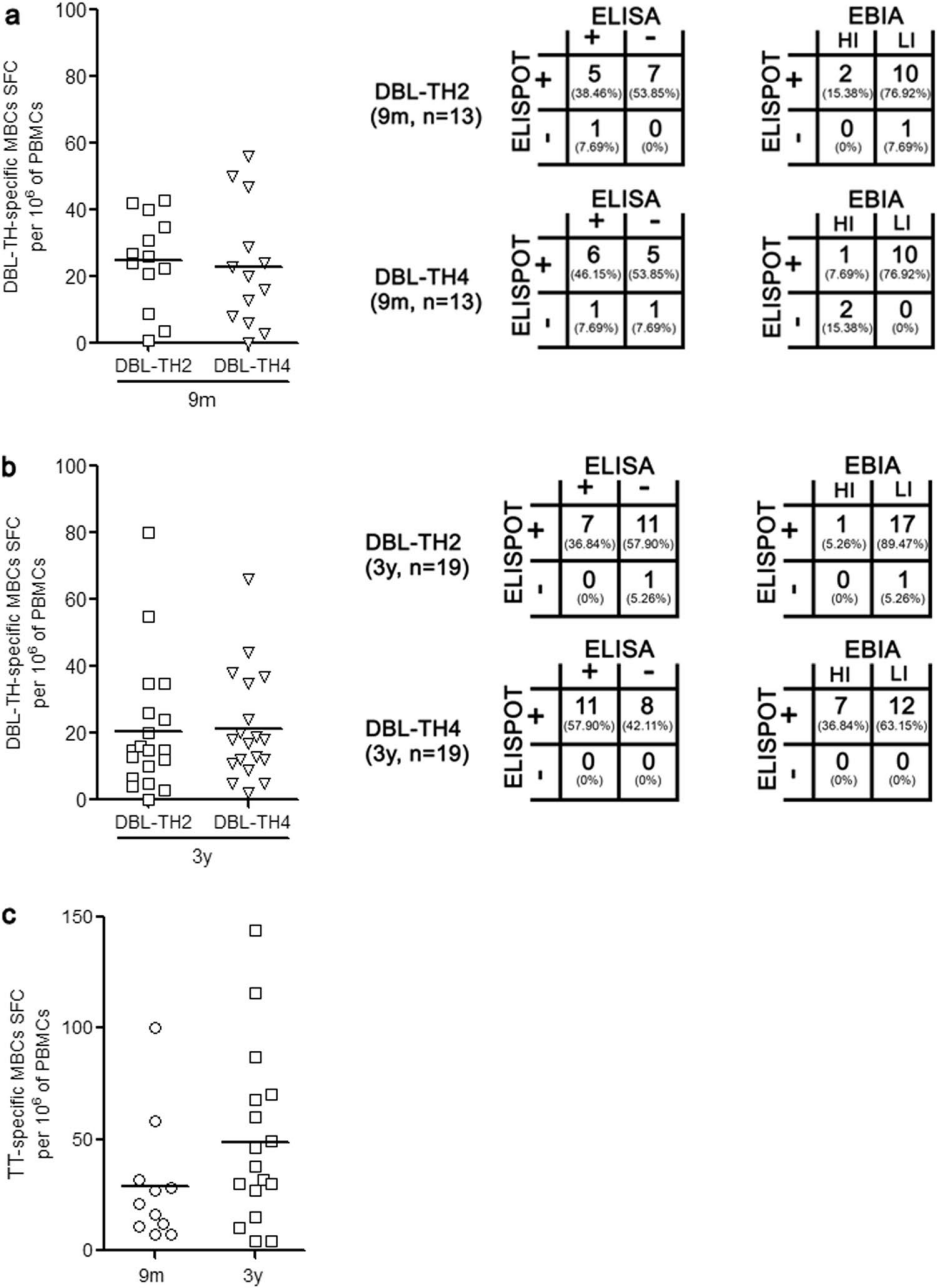
Long-lived DBL-TH-specific MBCs responses in individuals who had malaria attack for at least 3 years. MBC responses to a panel DBL-TH polymorphic strain were determined. PBMCs from 9 m and 3 y post-infection was used for ELISPOT assay. The scatter dot graph showing SFC-specific to DBL-TH2 and -TH4 per million stimulating PBMCs and the table showing association of plasma antibody response with ELISPOT specific to DBL-TH2 and -TH4 at (**a**) 9 m and (**b**) 3 y post-infection. HI refers to individual who had ≥ 80% inhibition activity of antibodies against DBL-TH-erythrocyte binding; LI refers to individual who had <80% inhibition activity. Tetanus toxoid-specific MBCs (**c**) was used as positive control of the experiment. The representative spot images are showed in Supplementary Fig. S3.

**Figure 6:**
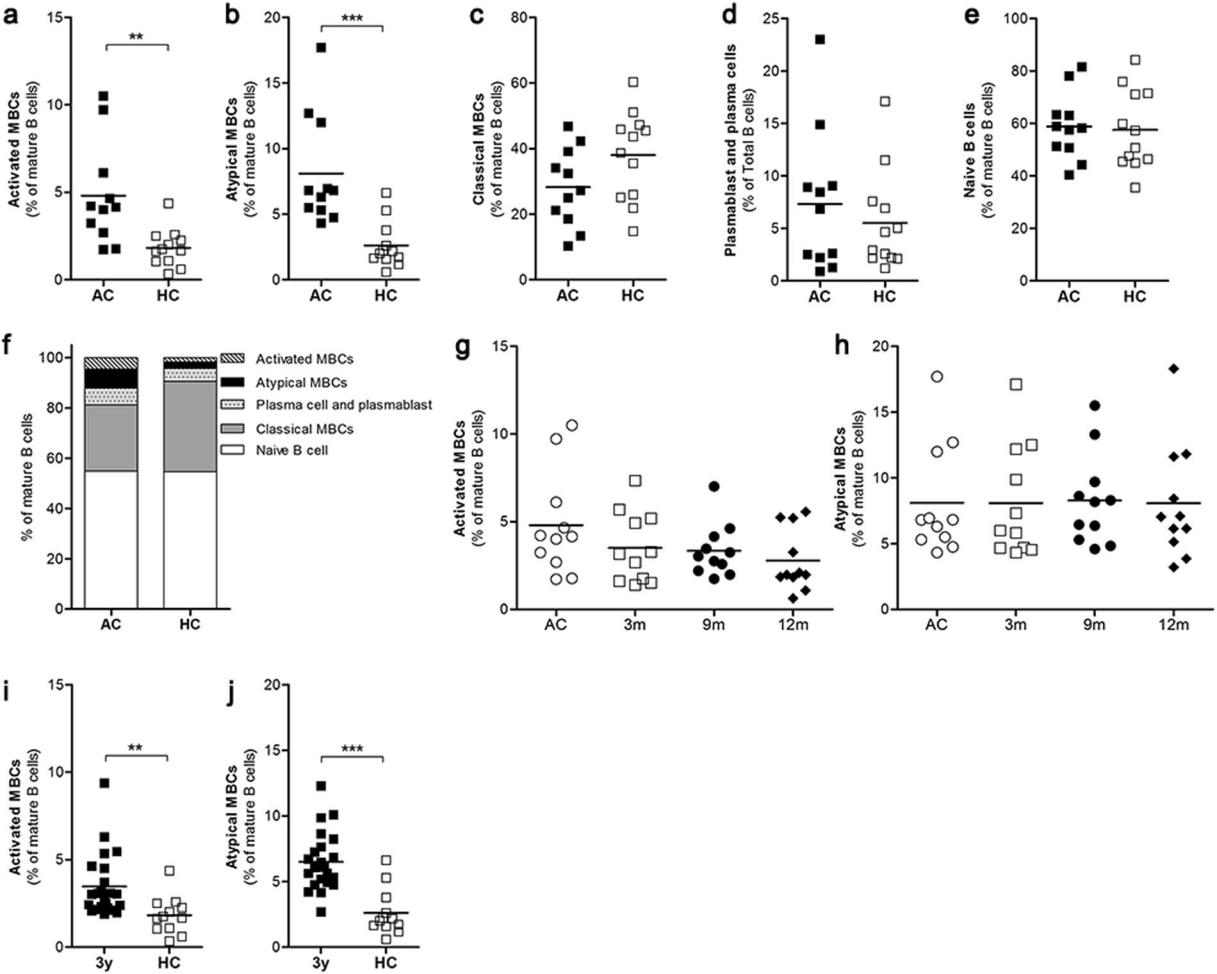
The expansion and persistence of activated MBCs and atypical MBCs. The frequencies of B cell subsets in individuals with acute *P. vivax* infection (AC, n = 11) compared to healthy controls (HC, n = 12) were quantified by flow cytometry. The total (**a**) activated MBCs (CD10^−^CD19^+^CD20^+^CD21^−^CD27^+^), (**b**) atypical MBCs (CD10^−^CD19^+^CD20^+^CD21^−^CD27^−^), (**c**) classical MBCs (CD10^−^CD19^+^CD20^+^CD21^+^CD27^+^), (**d**) plasmablasts and plasma cells (CD19^+^CD20^−^CD21^−^), (**e**) naïve B cells (CD10^−^CD19^+^CD20^+^CD21^+^CD27^−^) over the study periods. The relative percentages of the various B cell subsets in total mature B cells in each study group (**f**). The expansion of activated and atypical MBC was kinetically followed in individuals after recovery from infection at 3 m, 9 m and 12 m (**g**,**h**). The frequencies of activated and atypical MBCs in subjects 3 y post-infection (**i**,**j**). The grating strategies are presented in Supplementary Fig. S4.

**Figure 7:**
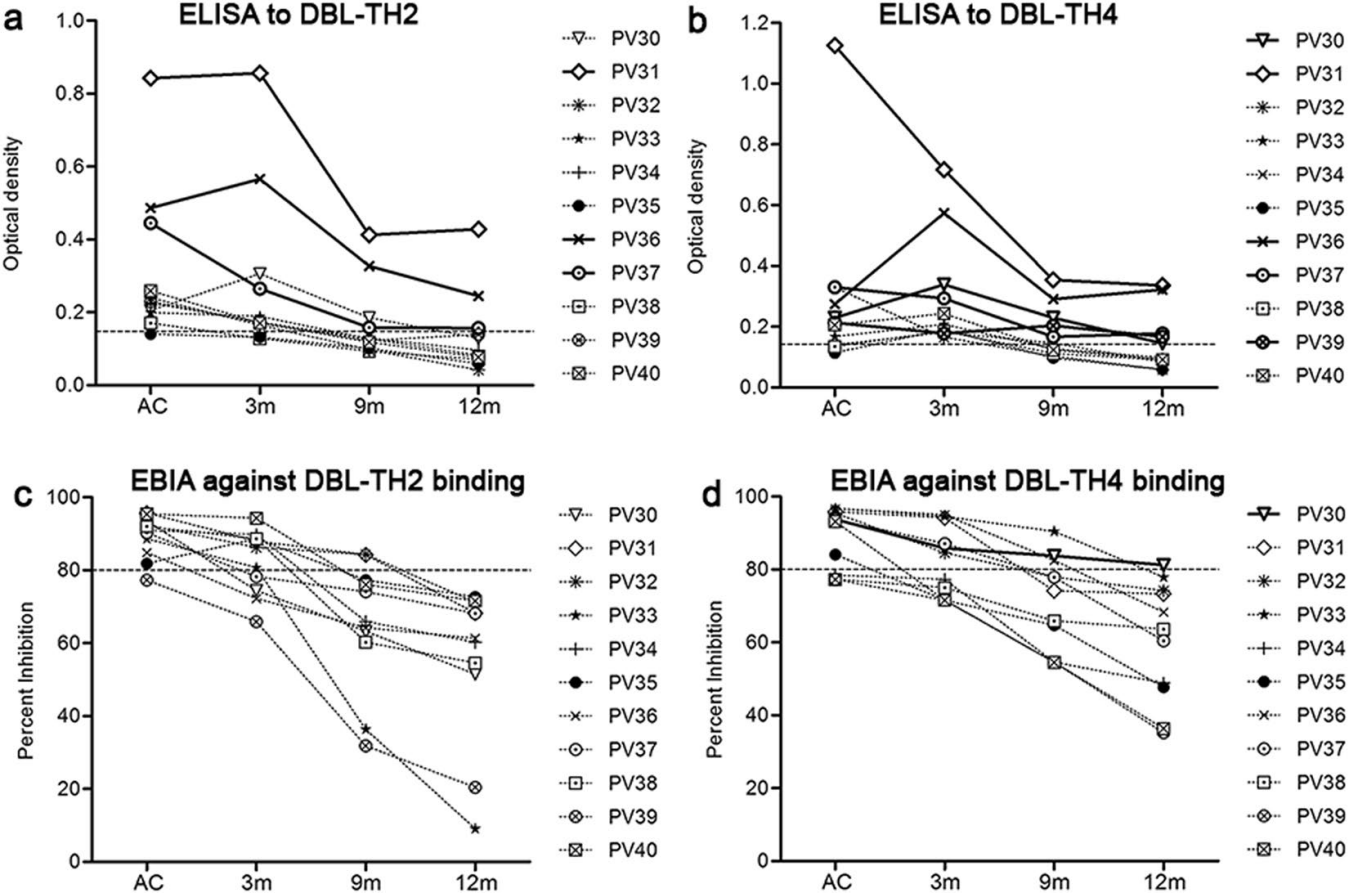
The association of activated MBC and atypical MBC expansion, antibody responses and DBL-TH-specific MBC frequency. The stability of titer levels and inhibitory function against erythrocyte binding of (**a**,**c**) anti-DBL-TH2 antibody and (**b**,**d**) anti-DBL-TH4 antibody.

Next, sub-cohort 2 was organized to test the hypothesis that polymorphic residues on DBL-TH may interfere persistence of long-lived MBCs. The 19 PBMC samples of *P. vivax* subject who had recruited in our study record when they were infected by *P. vivax* in the past 3 years were collected (Supplementary Table S4 and Fig. 5). The latest, sub-cohort 3 study was carried out in 11 individual samples for exploring the relation between longevity of antibody responses, DBL-TH-specific MBCs and the responses of B cell subset population in *P. vivax* infection. PBMCs were taken for B cell subset phenotyping, assessing kinetic of atypical and activated MBCs frequency during and post-infection and demonstrating the presence of DBL-TH-specific MBCs. Plasma sample was also used for evaluating persistence of DBL-TH antibody response (Figs 6 and 7).

### The antigenicity of DBL-TH variant antigens during and post-infection

To compare the antigenicity of a panel DBL-TH variants during acute and recovery phase of infection, IgG antibody titers specific to DBL-TH2, -TH4, -TH5, -TH6, -TH9 and reference Sal I strain were detected in AC patients and in 3 m, 9 m, 12 m post-infection. In AC patients, the percentage of positive responder to DBL-TH antigens and DBPII-Sal I was 97.50%, 92.50%, 92.50%, 97.50%, 77.50% and 85.00%, respectively (Fig. 2a). From seropositive patients, the prevalence of high responders to DBL-TH2, -TH4, -TH5, -TH6, -TH9 and DBPII-Sal I antigens were 95.00%, 77.50%, 85.00%, 72.50%, 35.00% and 50.00%, respectively (Fig. 2b). However, at post-infection, average antibody levels against DBL-TH2, -TH4, -TH5, -TH6, -TH9 and DBPII-Sal I antigens tended to decline overtime. The proportion of high responders at 9 m post-infection was significantly different in 27.11%, 34.48%, 31.03%, 20.69%, 27.59% and 13.79%, respectively, from the acute phase. The prevalence of high responders to those antigens was significantly decreased at later time points relative to the acute phase with 29.63%, 40.74%, 33.33%, 18.52%, 22.22% and 11.11% at 12 m after recovery (Fig. 2b). Thus, the kinetics of anti-DBL-TH responses were further explored in a longitudinal cohort study.

### Anti-DBL-TH responses was maintained up to 9 months after recovery

The stability of antibody responses was monitored for up to 12 m post-infection in patients classified as positive responders towards DBL-TH, using 19 samples from *P. vivax* subjects who had been able to follow up every time point of the cohort study. Seropositive patients displayed a significant positive titer to DBL-TH2, -TH4, -TH5, -TH6 and DBPII-Sal I at 9 m post-infection while antibody titer to DBL-TH9 variants persisted until 12 m (Fig. 3). Similarly, the inhibitory antibody activity against DBL-TH binding in these patients was maintained until 9 m post-infection. Monitoring inhibitory activity from positive responders who had HI function showed that approximately 58.82%, 73.33%, 27.27% and 58.33% of samples had the ability to maintain their inhibitory function against DBL-TH2, -TH4, -TH5 and -TH9 binding, respectively (Fig. 4a–d). Only some individuals maintained HI function at 12 m post-infection. The highest frequency of stable inhibition was shown in DBL-TH4 binding (60%) followed by DBL-TH9 (58.33%) and DBL-TH2 (47.06%). A much lower proportion of inhibition against erythrocyte binding was shown in DBL-TH5 with only 27.77% of the sample having stable HI function at 12 m post-infection.

### Long-lived DBL-TH-specific MBCs were detected at 3 years post-infection

As anti-DBL-TH antibody responses was significantly maintained at 9 m post-infection and high number of individuals had stable inhibition efficacy on antibody against DBL-TH2 and -TH4 binding. Thus, to explore the presence of DBL-TH-specific MBCs responses at recovery phase, DBL-TH2 and -TH4 were selected to answer this existing question. Of the total 19 vivax subjects, the 13 PBMC samples were available for ELISPOT assay. At 9 m post-infection, 12 (92.31%) and 11 (84.61%) samples had positive MBCs to DBL-TH2 and -TH4, respectively (Fig. 5a). However, when antibody responses were assessed in same patient, we found that the persistence of DBL-TH-specific MBCs was distinct from DBL-TH-specific antibody response. Among 12 positive DBL-TH2-ELISPOT samples, 5 samples (38.46%) were seropositive by ELISA and only 2 samples (15.38%) had high inhibitory function (Fig. 5a). For 11 samples of positive DBL-TH4-ELISPOT, 6 samples (45.45%) showed seropositive ELISA and only 1 samples (7.69%) had high inhibition (Fig. 5a). These observations suggest that at 9 m post-infection, despite levels and inhibition function of anti-DBL-TH antibody were lost, DBL-TH-specific MBCs was detectable in the absence of re-infection.

Thus, the presence of long-lived MBC responses to DBL-TH was further explored in 3 y post-infection subjects. The results showed that 18 (94.74%) individuals had detectable MBCs to DBL-TH2, and all individuals tested (100%) had detectable MBCs against DBL-TH4 (Fig. 5b). To correlate the persistence of long-lived MBCs response with antibody responses, all those 19 subjects was carried out to detect anti-DBL-TH titer. The results showed 7 samples (36.84%) were seropositive to DBL-TH2 antigen whereas only one sample (5.26%) maintained inhibitory antibody from the 18 positive ELISPOT samples (Fig. 5b). Of the 19 positive DBL-TH4-ELISPOT samples, 11 samples (57.90%) were seropositive to ELISA and 7 samples (36.84%) had an effective inhibitory antibody (Fig. 5b). These data confirm that the persistence of antibody response and inhibitory activity against DBL-TH were independently associated with stable frequencies of long-lived DBL-TH-specific MBCs. As expected, almost all subjects, 86.86%, had MBCs to tetanus toxoid antigen since it is commonly used as a routine vaccine for Thai residents (Fig. 5c).

### The expansion of activated MBCs and atypical MBCs during and after recovery from infection

To identify which B cell populations that may be associated with antibody and MBCs specific to DBL-TH at post-infection, first we detected the B cell subsets that responded during acute malaria infection. All AC had significantly expanded activated MBC and atypical MBC populations compared to HC (Fig. 6a,b). However, the frequencies of classical MBCs, plasmablast/plasma cells and native B cells in AC were not significantly different from HC (Fig. 6c–f). Thus, the expansion of activated and atypical MBCs was observed by follow them for 12 m post-infection. The results show that activated MBC and atypical MBC were stably detectable in all subsequent follow up times showing no significance difference of those frequency at 3 m, 9 m and 12 m compared to AC (Fig. 6g,h). As an additional support, we analyzed B cell subsets from 3 y post-infection individuals who showed positive DBL-TH-specific MBCs by ELISPOT. We found that the frequencies of activated and atypical MBCs were significantly high after at least 3 years since last malaria attack when comparing HC (Fig. 6i,j).

### The expansion of activated MBCs and atypical MBCs was related to short-lived anti-DBP responses

Expansion of atypical MBCs has been demonstrated in the inhibition of immunoglobulin production in *P. falciparum* malaria^22^. Thus, antibody and MBC response in the individual’s patients who had expansion of activated and atypical MBCs was evaluated. As for antibody titers, anti-DBL-TH2 antibody titers in seven samples from a total of 11 infected patients had obviously declined at 9 m post-infection. Only three patients maintained seropositivity to DBL-TH2 at 12 m post-infection (Fig. 7a). More samples, five from the total, had stable anti-DBL-TH4 responses at 12 m post-infection (Fig. 7b). As for stability of inhibitory antibodies against erythrocyte binding, none of the 11 infected patients had stable inhibitory anti-DBL-TH2 antibodies, and only 1 individual had stable anti-DBL-TH4 binding to erythrocytes at 12 m post-infection (Fig. 7c,d).

In addition, all *P. vivax* subjects who had increased activated and atypical MBCs population maintained a positive number of DBL-TH2 and -TH4-specific MBCs at 9 m post-infection. The 11 subjects (100%) had DBL-TH2-positive MBCs and 9 samples (81.82%) were positive to DBL-TH4, implying those MBCs appear closely related to the development of DBL-TH-specific MBCs. Taken together, these findings suggest that the expansion of activated and atypical MBCs does not interfere long-lived MBCs specific to DBL-TH but appear to be related to short-lived anti-DBL-TH antibody responses.

## Discussion

In this study, it is important to note that in situations of low exposure to *P. vivax* parasite, an acquired long-lived MBC specific to polymorphic strains of DBL-TH was produced and stably maintained in the absent of re-infection. There was expansion of activated and atypical MBCs during infection and an expansion of these two MBC subset stably persisted at post-infection. The persistence of activated and atypical MBCs was related to short-lived anti-DBL-TH response whereas it was not affect to the maintenance of DBL-TH-specific MBCs at post-infection.

Our findings demonstrate high antigenicity of a panel of DBL-TH variant antigens compared to reference strain Sal I. High antibody titers and strong inhibitory antibodies were produced during *P. vivax* infection, preferentially to variant strains. These antibodies maintained a positive ELISA response and inhibitory function against DBL-TH-erythrocyte binding until 9 m post-infection without re-exposure. Only for some patients sustainably produced antibody against DBL-TH variants at 12 m post-infection, indicating that patients are able to produce and maintain antibody response to polymorphic strains of DBPII in low malaria endemicity. This finding support the results of prior studies in which the persistence of antibody responses to blood-stage malaria antigens was persisted for more than 6 years, 37 months and 5 months after their last malaria episode in patients living in areas of low malaria transmission in Northern Thailand, Northwestern Brazil and Southern Peru, respectively^16,20,23^. However, our data underline the need for a larger sample size to obtain confidence in our estimation of the persistence of inhibitory antibody and its association with reduced risk of clinical vivax malaria.

In malaria research, patients who lived in low malaria transmission areas had more effective antibody and MBCs responses^13,16,20,23^ whereas some studies in high transmission areas showed short-lived antibody and inefficient MBCs responses^17^, particularly in younger individuals. We found detectable frequencies of MBCs specific to polymorphic strains of DBPII, DBL-TH2 and -TH4, in majority of adult subjects at 9 m and 3 y post-infection. However, few samples whose positive for ELISPOT have high anti-DBL-TH antibodies and long-lasting functionally inhibitory antibody. These results indicate that DBL-TH-specific MBCs were efficiently induced by primary *P. vivax* infection and sustained longer than serum antibody responses. It is possible that, in agreement with a previous study^15,24^, DBL-TH-specific MBCs can be maintained over long time periods in the apparent absence of antigen while the pathogen’s replication rate is required for proliferation and differentiation of MBCs into antibody-secreting cell (ASC) to keep serum antibody levels constant. Alternatively, MBCs could be differentiated into impaired MBCs, which may contribute to the inefficient acquisition of antibody production.

It has been described in high malaria transmission areas that chronic exposure to *P. falciparum* can lead to the expansion of atypical MBCs. This MBCs subset expressed FCLR5 inhibitory receptors that play a role in interfering with immunoglobulin production compared to classical MBCs^14,22^. Consistent with the observation of febrile malaria children in Mali, malaria induces the activation of Th1 cytokines and Tfh-1 cells that contribute to the expansion of T-bet^hi^ atypical MBCs, also causing reduced BCR signaling^25^. The study of development of MBCs in newborns and mother in malaria endemic area in Uganda showed an expansion of atypical MBCs and non-IgG ^+^ MBCs which increased with age^26^. In a low malaria transmission area in Brazil, found that single- and multiple- *P*. *vivax*-infected individuals reveal higher frequencies of atypical MBCs and lower proportions of classical MBCs. These atypical MBCs were decreased after treatment for 35 days whereas frequency of classical MBCs and antigen-specific antibody levels were increased^27^. Here, our results showed that *P. vivax* infection maintained an expansion of activated and atypical MBC populations. It was related with the development of DBL-TH-specific MBCs as all individuals who had these two MBC expansion showed positive ELISPOT at 9 m after recovery. Moreover, number of re-exposure also seem to link with frequencies of activated and atypical MBCs as one subject who had highest percentage of those MBCs subset had re-infection during following (Supplementary Table S4). Interestingly, we observed an alteration of the ratio between activated MBCs and atypical MBCs from each individual. This ratio was increased from 1:2 at acute phase to 1:3, 1:3 and 1:4 at 3 m, 9 m and 12 m post-infection, respectively. A possibility is that while undergoing *P. vivax* infection, activated MBCs were triggered by vivax parasites and this was sufficient to promote ASC to secret high levels of antibody. After recovery from an infection, in the absence of antigen, it is likely that ASC are short-lived and constantly replaced by atypical MBCs, although the activated MBCs still remain in blood circulation. However, the expansion of atypical MBCs in the recovery phase occurred to be linked with instability of antibody responses due to only a few subjects from the total positive ELISPOT samples having positive antibody titers and functional inhibitory antibody at 9 m post-infection. One explanation for these observations might be that expansion of atypical MBCs was an evasive mechanism of the parasite to suppress MBC function as we have assumed above. Further study is needed to demonstrate the MBC subset that plays a role in maintenance of efficient antibody responses to DBL-TH.

A limitation of this study is insufficiency to clear implication of the correlation between atypical MBCs expansion and its interference on antibody responses to malaria antigen. Given our inability to recruit a large sample size of *P. vivax* subject in one-year cohort study, 11 patients had been able to follow. This observation might be only tentative result to demonstrate that circulating atypical MBCs from *P. vivax* malaria infected patients are stably presence in whom who had short-lived anti-DBL-TH antibodies, it may not represent the fully understand the biology of these cell in response to DBL-TH variant antigens. Nonetheless, this finding was consistent with previous studies^16,22,27–31^ conducted in other malaria endemic areas which is mostly have been focus on AMA1 and MSP1-19 antigens. Our study represents the first report of immunological memory specific to polymorphic haplotypes of DBPII by observing the association of antibody levels, inhibition function and MBCs as well as an alteration of activated MBCs/atypical MBCs during recovery from *P. vivax* infection. It is therefore reassuring that although DBL-TH-specific antibodies declined to seronegative levels and lost functional inhibition in the absence of persistent *P. vivax* exposure, their DBL-TH-specific MBCs were long-lived at least 3 years after their last malaria episode.

This study concludes that antibody and MBCs responses to polymorphic strains of DBPII were induced during natural *P. vivax* infection under the condition of low malaria transmission. Over recovery phase, majority of patients were unable to maintain an efficacy of anti-DBL-TH antibody response as antibody levels and inhibitory function were significantly declined at 9 m post-infection. However, DBL-TH specific MBCs were longer detected in greater than 80% of individuals who recovered from infection for 9 m and 3 y. The persisting of activated and atypical MBC displayed to be related with longevity of DBL-TH-specific MBCs, whereas patients who showed expansion of activated and atypical MBC had short-lived anti-DBL-TH responses. Further research is necessary to understand the profiling function of DBL-TH-specific atypical MBC and its effect on humoral immunity in malaria with long- or short-term parasite exposure. This knowledge will be useful for the development of a novel strategy to generate and enhance long-lasting humoral immunity to malaria.

## Methods

### Ethical Statement

This study was approved by the Ethic Committee on Human Rights Related to Human Experimentation, Mahidol University (MUIRB2012/079.2408). Before recruitment, the consent form was provided and explained about the experimental procedure in the language best understood to all study participants. Written informed consent was obtained from each study participant. The experiments involving human subject were conducted in accordance with relevant guidelines and regulation.

### Study area and sample collection

In this study, both cross-sectional survey and cohort study were designed to demonstrate the antigenicity of polymorphic DBL-TH strain in induction of antibody and MBC response in malaria endemic areas, Rap Ro Village, Tha Sae, Chumphon Province which is located in southern Thailand where *P. falciparum* and *P. vivax* are the common parasites (Fig. 1). In cross-sectional survey, between May 2014-May 2017, the prevalence of anti-DBL-TH response during and post-infection was observed in symptomatic *P. vivax* patients (AC, n = 40) and after the patients recovered from infection for 3 months (3 m, n = 36), 9 months (9 m, n = 29) and 12 months (12 m, n = 27).

For the cohort study, 3 sub-cohorts were taken into this study. The sub-cohort 1 was carried out from May 2014 to May 2017 to explore the longevity of anti-DBL-TH antibody (n = 19) and DBL-TH specific MBC responses (n = 13). The sub-cohort 2 was organized from subjects who had experienced to *P. vivax* in past 3 year, since 2014, to examine long-lived DBL-TH-specific MBCs (3 y, n = 19). In sub-cohort 3, the samples were collected in 2016–2017 for studying the relation between the longevity of anti-DBL-TH responses, DBL-TH-specific MBCs and the expansion of atypical and activated MBC frequency post-infection (n = 11).

Confirmation of *P. vivax* infection was performed by microscopic examination of thin and thick Giemsa-stained blood smears and by nested PCR. We survey the data of past-infection or no. of prior infection to parasite in individuals from the record of Vector borne Disease Unit. The patients were scheduled to collect blood sample in every 3 months and to check for sub-patent malaria. To estimate the incidence of clinical malaria over the study period, malaria staff had carried out weekly house to house visit from May 2014-May 2017

Healthy controls (HC, n = 60) was collected from individuals who had no history of malaria infection.

### IgG antibody responses by ELISA

Plasma samples from *P. vivax*-exposed and healthy control individuals were tested by ELISA for antibody response against the panel of DBL-TH compared with reference strain Sal I. Six recombinant proteins (Supplementary Table S1), DBL-TH2, -TH4, -TH5, TH6, and -TH9, and DBPII Sal I at 2 µg/mL were coated, blocked with 5% skim milk in PBS-T and incubated with plasma at a dilution of 1:200. The plates were washed and horseradish peroxidase conjugated goat anti-human IgG secondary antibodies were added into the wells, incubated at RT for 1 hour. Following addition of ABTS substrate, absorbance at 405 nm was measured. A seropositive was considered an OD value greater than the cut-off value, which is calculated as the average OD from 60 healthy controls + 2 SD. Seronegative was considered as a none responder (NR), which had an OD value lower than cut-off. To categorize the intensity of positive response, responses were classified into 2 groups. A result greater than or equal to the cut-off + 2 SD was classified as “high responder” (HR) and a result greater than the cut-off but less than the cut-off + 2 SD was classified as “low responder” (LR).

### COS7 cell erythrocyte binding inhibition assay

The longevity of inhibitory function of neutralizing antibodies to inhibit the binding of polymorphic DBL-TH and DBPII Sal I to human erythrocytes were evaluated by COS7 EBIA as described elsewhere^32^. Briefly, plasma were diluted at 1:100 dilution and pre-incubated with DBPII-transfected COS7 cells at 37 °C for 1 hour. Then 10% Duffy positive erythrocytes were added and incubated further 2 hours. Quantifying rosettes by counting over 30 fields of view at 20 × magnification. The inhibitory function was calculated as the percentage of rosettes in the presence of patient plasma divided by those in the presence of negative control plasma. The EBIA experiment was done in triplicate wells for each DBL-TH variant and repeated two times. The inhibition activity in individuals was classified into 2 groups: “high inhibition” (HI) was considered inhibition greater than or equal to 80% and inhibition less than 80% was classified as “low inhibition” (LI).

### ELISPOT assay

PBMCs were isolated, washed and checked cell viability by trypan blue exclusion test. PBMCs were adjusted to a concentration of 1 × 10^6^ cell/mL and distributed into 24-well culture plates. The cells were stimulated with a mixture of 1 µg/mL of R848 and 10 ng/mL of rhIL-2 (Mabtech, Stockholm, Sweden). Cells were cultured under 5% CO_2_ at 37 °C for 3 days.

The 96-well ELISPOT plates (Millipore) were coated with 10 µg/mL of rDBL-TH or 15 µg/mL of mAb MT91/145 in PBS at 4 °C overnight. The wells were blocked with R10 (10% FBS in RPMI 1640 media) at RT for 1 hour. Stimulated PBMCs were harvested, washed and incubated in DBL-TH antigen-coated ELISpot wells for 16–24 hours. The wells were washed and incubated with 100 µL of 1 µg/mL mAb MT78/145 at RT for 2 hours. After washing with PBS, 100 µL of Streptavidin-conjugated HRP was added and incubated at RT for 1 hour. The plate was washed and 100 µL of TMB substrate solution was used to detect the spot. After 10 mins, the enzymatic reaction is stopped by rinsing the plate with deionized water. Spots were count by Bioreader 5000 Pro-F gamma ELISPOT Reader (BioSys GmbH, Germany) (Supplementary Fig. 3). The positive response of antigen-specific MBCs was defined as spot-forming cells (SFC) with 2-fold higher total number of spots relative to the negative control.

### B cell subset phenotyping

PBMCs from during and post-*P. vivax* infection were used for B cell subset phenotyping. All B cell phenotypic analyses were performed using mouse monoclonal antibodies (mAbs) specific for human B cell markers conjugated to fluorophores as follows: FITC-CD19, PerCP-CD20, PE/Cy7-CD10, PE-CD27, and APC-CD21 (Biolegend). Using this strategy as showed in Supplementary Fig. 4, we observed the proportion of naive B cells as the number of CD10^−^CD19^+^CD20^+^CD21^+^CD27^−^cells; plasma cells/blasts were of CD19^+^CD21^−^CD20^−^ cells; immature B cells were CD19^+^CD10^+^ cells; classical MBCs were CD10^−^CD19^+^CD20^+^CD21^+^CD27^+^ cells; atypical MBCs were of CD10^−^CD19^+^CD20^+^CD21^−^CD27^−^ cells; and activated MBCs were CD10^−^CD19^+^CD20^+^CD21^−^CD27^+^ cells. The relative proportions of all the B cell subpopulations as analyzed per total CD19^+^ B cells for each sample group was determined. FACS analyses were performed on a FACSCanto II flow cytometer (BD Biosciences) using FlowJo software (Tree Star).

### Statistical analysis

The differences in antibody response to different DBL-TH allele or DBPII-Sal I among independent groups (AC, 3 m, 9 m, 12 m post-infection and HC), as well as the difference in subsets of B cell between acute patients and healthy controls were compared by Mann–Whitney *U* test. Wilcoxon signed rank test was used to analyze stability of antibodies or subset of MBCs in each individual for each time point post-infection compared to acute phase. In all analyses, 2-tailed *P*-value < 0.05 was considered significant. The statistical analysis was performed and graphs were prepared using GraphPad Prism (GraphPad Software, USA).

## Electronic supplementary material

Supplementary information
