# Supplementary material for: Persistence of Long-lived Memory B Cells specific to Duffy Binding Protein in individuals exposed to Plasmodium vivax
Source: Sci Rep. 2018 May 29;8:8347. doi: 10.1038/s41598-018-26677-x (PMC5973932; doi:10.1038/s41598-018-26677-x)
Supplement: Supplementary file 1 — Supplementary information [file 41598_2018_26677_MOESM1_ESM.docx]

**Persistence of Long-lived Memory B Cells specific to Duffy Binding Protein in individuals exposed to *Plasmodium vivax***

Siriruk Changrob^1^, Amy M. McHenry^2^, Myat Htut Nyunt^3^, Jetsumon Sattabongkot^4^, Eun-Taek Han^3^, John H. Adams^5^, and Patchanee Chootong^1^*

^1^Department of Clinical Microbiology and Applied Technology, Faculty of Medical Technology, Mahidol University, Bangkok, 10700, Thailand.

^2^Department of Biological Sciences, Southwestern Adventist University, Keene, Texas, 76059, USA.

^3^Department of Medical Environmental Biology and Tropical Medicine, School of Medicine, Kangwon National University, Chuncheon, Gangwon-do, 200-701, Republic of Korea.

^4^Mahidol Vivax Research Unit, Faculty of Tropical Medicine, Mahidol University, Bangkok, 10400, Thailand.

^5^Department of Global Health, University of South Florida, Tampa, Florida, 33612, USA.

*Corresponding author: Patchanee Chootong. Faculty of Medical Technology, Mahidol University (Salaya Campus), 999 Phutthamonthon 4 Road Salaya, Phutthamonthon, Nakhon Pathom 73170, Thailand. Tel: (+66) 2411 1096, Fax: (+66) 2411 4110

Email: pchooton@gmail.com

**Supplementary information**

**Recombinant DBL-TH protein production.** The recombinant DBL-TH proteins, corresponding to previous study of the *P. vivax* DBP among Thai isolates, were expressed in *E. coli^1-3^*. DNA fragments encoding the DBL-TH2, -TH4, -TH5, -TH6 and –TH9 haplotype (Supplementary Table S1) were constructed in the pEGFP plasmid by site-directed mutagenesis using DBPII Sal I as template. The DNA fragments were amplified by PCR and cloned into pET23a^+^ vector in BL21 (DE3) *E. coli*. The bacterial clones expressing the recombinant proteins were expressed and induced with 1 mM of isopropyl-β-D-thiogalactopyranoside (IPTG) for 6 hours. The inclusion bodies were solubilized, purified under denaturing conditions and processed for refolding for the recovery of properly folded structures as previously described^4^. The refolded proteins were dialyzed and purified by iron exchange chromatography using HiTrap SP FF column (GE Healthcare, USA). The purified recombinant DBL-TH antigen was evaluated for purity, homogeneity, and conformation by sodium dodecyl sulfate (SDS)-polyacrylamide gel electrophoresis (PAGE) under reducing and non-reducing conditions and immunoblot analysis.

**Expression, purification and Western blot analysis of DBL-TH proteins.** The recombinant proteins encoding DBL-TH2, -TH4, -TH5, -TH6 and -TH9 were successfully expressed and purified under denaturing conditions, and refolded by rapid dilution. The mobility shift of refolded and denatured antigens was used to demonstrate the presence of disulfide formation in the native conformation of recombinant antigens. Refolded rDBL-TH antigens migrated as a single band at the expected mass of ~37 kDa compared to denatured antigens at ~39 kDa on SDS-PAGE (Supplementary Fig. S1). To confirm the presence of His-tagged recombinant proteins, immunoblots probed with an anti-His tag antibody and anti-DBPII antibody (mAb-3D10) were used to illustrate the recognition of rDBL-TH (Supplementary Fig. S2).


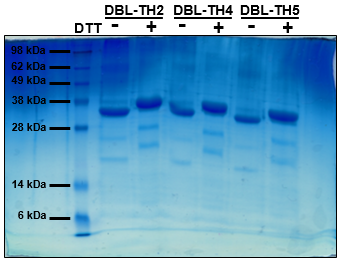

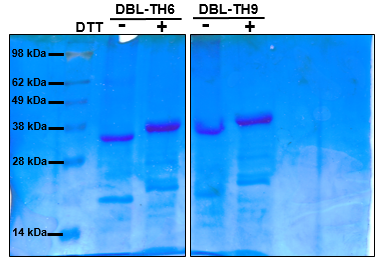


**Supplementary Figure S1.** Production of refolded DBL-TH proteins. The recombinant protein encoding DBL-TH2, -TH4, -TH5, -TH6 and -TH9 were successfully expressed and purified under denaturing conditions, and refolded by rapid dilution. The mobility shift of refolded on SDS-PAGE gel before (-) and after (+) reduction with DTT was used to demonstrate the presence of disulfide formation in the native conformation of recombinant antigens. Refolded rDBL-TH antigens migrated as a single band at the expected mass of ~37 kDa compared to denatured antigens at ~39 kDa on SDS-PAGE.


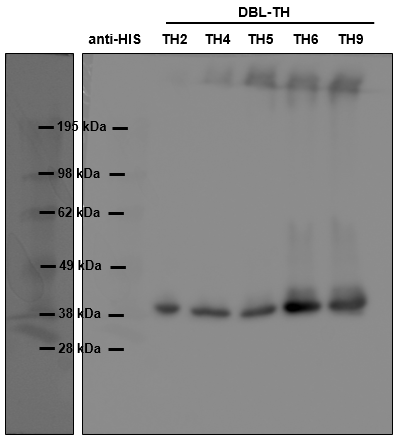

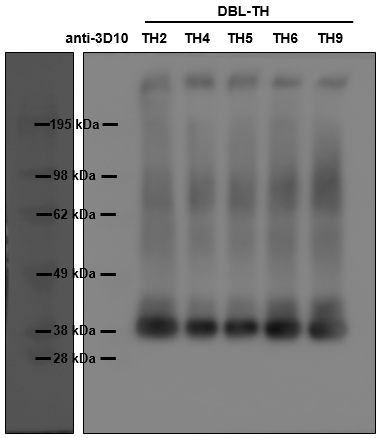
 **Supplementary Figure S2.** Immunoblotting of DBL-TH protein. To confirm the presence of His-tagged recombinant proteins, immunoblots probed with an anti-His tag antibody and anti-DBPII antibody (mAb-3D10) were used to illustrate the recognition of rDBL-TH.

**
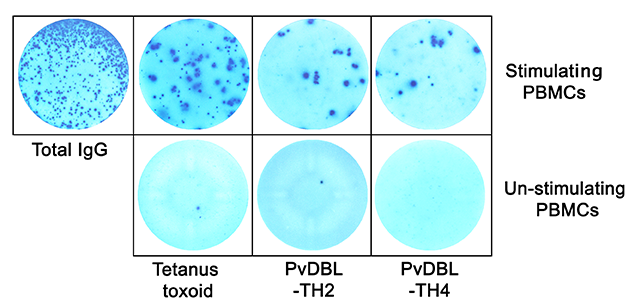
**

**Supplementary Figure S3.** DBL-TH specific MBC response by ELISPOT assay. DBL-TH-specific MBCs responses were revealed by ELISPOT 3 days after polyclonal stimulation. ELISPOT plates were coated with recombinant DBL-TH2, -TH4, TT and anti-human IgG antibody to enumerate antigen-specific SFU and total IgG-secreting B-cells, respectively, For the analysis of antigen specific MBCs responses, PBMCs were cultured with or without stimulation of R848 and recombinant human IL-2. After 3 days, cells were harvested and applied to ELISPOT assay.


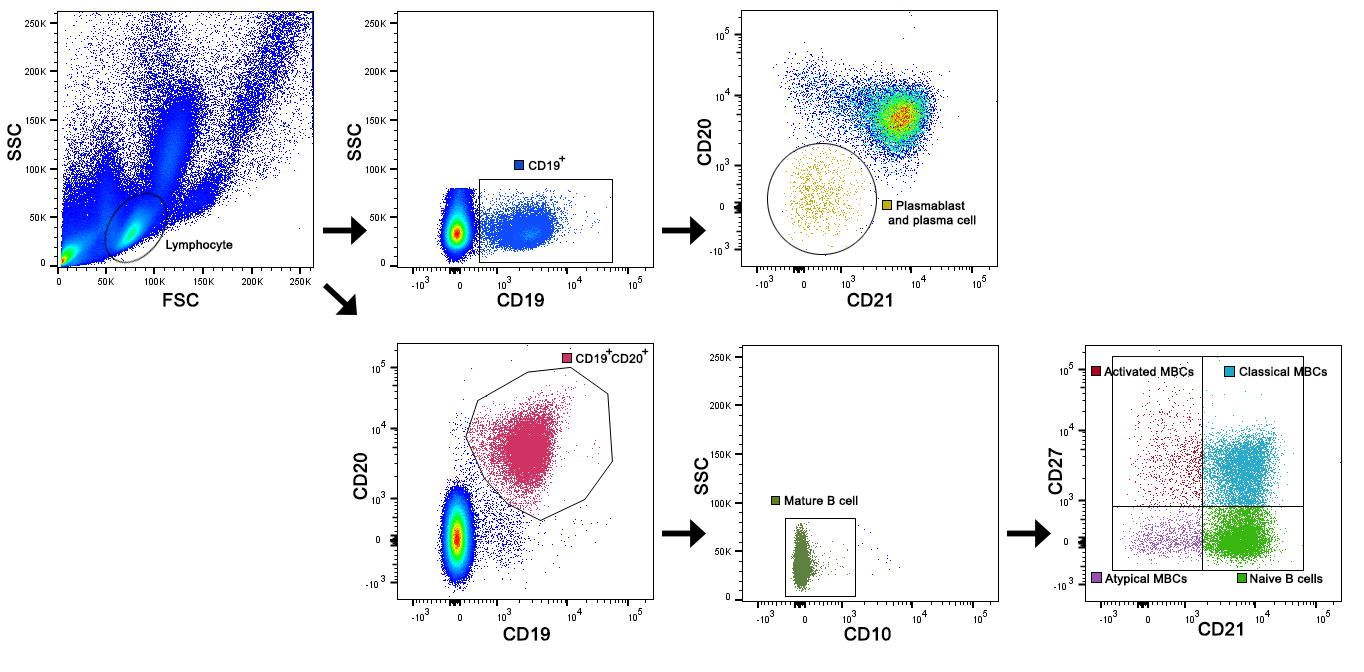


**Supplementary Figure S4.** Flow cytometry gating strategies for B cell phenotyping. FACS plots of B cell subsets of a representative malaria naïve Thai resident and a *P. vivax*-exposed volunteer. Within the CD19^+^CD20^+^ gate, the B cell subpopulations are defined as follows: Activated MBC (blue) (CD10^-^CD27^+^CD21^-^), Classical MBC (purple) (CD10^-^CD27^+^CD21^+^), Atypical MBC (red) (CD10^-^CD27^-^CD21^-^), Naive (green) (CD10^-^CD27^-^CD21^+^), and Plasma cells or plasmablast (orange) (CD19^+^CD21^-^CD20^-^)

**References**

1 Wongkidakarn, S., McHenry, A. M., Sattabongkot, J., Adams, J. H. & Chootong, P. Strain-Transcending Inhibitory Antibodies against Homologous and Heterologous Strains of Duffy Binding Protein region II. *PloS one* **11**, e0154577, doi:10.1371/journal.pone.0154577 (2016).

2 Chootong, P. *et al.* Characterization of inhibitory anti-Duffy binding protein II immunity: approach to Plasmodium vivax vaccine development in Thailand. *PloS one* **7**, e35769, doi:10.1371/journal.pone.0035769 (2012).

3 Chootong, P., McHenry, A. M., Ntumngia, F. B., Sattabongkot, J. & Adams, J. H. The association of Duffy binding protein region II polymorphisms and its antigenicity in Plasmodium vivax isolates from Thailand. *Parasitology international* **63**, 858-864, doi:10.1016/j.parint.2014.07.014 (2014).

4 Singh, S. *et al.* Biochemical, biophysical, and functional characterization of bacterially expressed and refolded receptor binding domain of *Plasmodium vivax* duffy-binding protein. *The Journal of biological chemistry* **276**, 17111-17116, doi:10.1074/jbc.M101531200 (2001).

**Supplementary Table S1:** Panel of DBL-TH alleles used for protein expressions. Polymorphic residues within DBPII and positions with reference to DBPII-Sal I (bold) are indicated. Conserved residues are represented by a dot (.).

| **DBPII alleles** | **Amino acid position** | | | | | | | | | | | | | | |
| --- | --- | --- | --- | --- | --- | --- | --- | --- | --- | --- | --- | --- | --- | --- | --- |
|  | **308** | **313** | **333** | **371** | **375** | **384** | **385** | **386** | **390** | **417** | **424** | **433** | **437** | **475** | **503** |
| **DBPII-Sal I** | **R** | **.** | **L** | **K** | **.** | **D** | **E** | **K** | **R** | **N** | **L** | **.** | **W** | **P** | **I** |
| DBL-TH2 | . | . | F | E | . | G | K | Q | . | K | I | . | R | . | . |
| DBL-TH4 | . | . | F | . | . | G | K | Q | H | K | I | . | R | . | K |
| DBL-TH5 | . | . | . | E | . | G | . | N | . | K | I | . | R | . | K |
| DBL-TH6 | . | . | . | . | . | G | . | H | . | . | . | . | . | . | . |
| DBL-TH9 | . | . | F | . | . | . | . | . | . | . | I | . | R | . | K |

**Supplementary Table S2:** Characteristic of study participants in survey of DBL-TH antigenicity at different time point after *P. vivax* infection.

| **Characteristic** | ***P. vivax*-exposed subjects** | | | | **Healthy subject** |
| --- | --- | --- | --- | --- | --- |
|  | **Acute vivax patients** | **Recovered subjects** | | |  |
|  |  | **3 months** | **9 months** | **12 months** |  |
| **Total (n)** | 40 | 36 | 29 | 27 | 60 |
| **Sex - no. (%)** |  |  |  |  |  |
| Male | 28 (70) | 25 (69.4) | 17 (58.6) | 18 (66.7) | 19 (31.7) |
| Female | 12 (30) | 11 (30.6) | 12 (41.4) | 9 (33.3) | 41 (68.3) |
| **Age - yr.** |  |  |  |  |  |
| Mean ± SD | 37.4 ± 12 | 38.2 ± 13.2 | 39.9 ± 12.2 | 40.1 ± 12.3 | 20.5 ± 1.3 |
| Range | 18-63 | 18-63 | 18-63 | 18-63 | 19-22 |
| **Parasitaemia (parasite/µL)** |  |  |  |  |  |
| Mean ± SD | 5170.8 ± 5238.3 |  |  |  |  |
| Range | 200-15,000 |  |  |  |  |

**Supplementary Table S3:** Age and reported number of prior *P. vivax* exposure in longevity antibody response study.

| **By antigen** | **DBL-TH antibody longevity study participants characteristic (n=19)** | | | | | | |
| --- | --- | --- | --- | --- | --- | --- | --- |
|  | **No. of positive responder at acute** | **Age** | **Sex** | | **No. of prior exposures** | | |
|  |  | **Mean ± SD** | **Male** | **Female** | **0** | **1** | **>1** |
| DBL-TH2 | 18 | 41.9 ± 12.4 | 13 | 5 | 17 | 1 | 0 |
| DBL-TH4 | 16 | 42.9 ± 11.3 | 12 | 4 | 15 | 1 | 0 |
| DBL-TH5 | 16 | 42.4 ± 12.7 | 11 | 5 | 15 | 1 | 0 |
| DBL-TH6 | 18 | 41.9 ± 12.4 | 13 | 5 | 17 | 1 | 0 |
| DBL-TH9 | 12 | 45 ± 11.8 | 8 | 4 | 11 | 1 | 0 |
| DBPII Sal I | 16 | 42.4 ± 12.7 | 11 | 5 | 15 | 1 | 0 |

**Supplementary Table S4:** Age and reported number of prior *P. vivax* exposure in DBL-TH specific MBCs response study participants

| **ELISPOT** | **Recovery phase** | **Age** | **Sex** | | **No. of prior exposures** | | | | **No. of recorded of re-infection** |
| --- | --- | --- | --- | --- | --- | --- | --- | --- | --- |
|  |  | **Mean ± SD** | **Male** | **Female** | **0** | **1** | **>1** | **NA^a^** |  |
| 9 months post-infection | DBL-TH2 MBCs |  |  |  |  |  |  |  |  |
|  | Positive | 40.4 ± 12 | 9 | 3 | 10 | 1 | 0 | 1 | 2 |
|  | Negative | 29 | 1 | 0 | 0 | 0 | 0 | 1 | - |
|  | DBL-TH4 MBCs |  |  |  |  |  |  |  |  |
|  | Positive | 38.3 ± 10.4 | 8 | 3 | 11 | 0 | 0 | 0 | 0 |
|  | Negative | 49.5 ± 19.1 | 2 | 0 | 1 | 1 | 0 | 0 | 0 |
| 3 years post-infection | DBL-TH2 MBCs |  |  |  |  |  |  |  |  |
|  | Positive | 42.9 ± 14 | 9 | 9 | 18 | 0 | 0 | 0 | 2 |
|  | Negative | 18 | 0 | 1 | 1 | 0 | 0 | 0 | 0 |
|  | DBL-TH4 MBCs |  |  |  |  |  |  |  |  |
|  | Positive | 41.4 ± 15 | 9 | 10 | 19 | 0 | 0 | 0 | 2 |
|  | Negative | - | - | - | - | - | - | - | - |

^a^ NA, not available

**Supplementary Table S5:** Characteristics of study participants in kinetic study of atypical MBCs.

| **Participants’ characteristic (n=11)** | **Mean or Number** | **Range** |
| --- | --- | --- |
| Age (years) | 40.4 | 51-18 |
| Sex |  |  |
| Male | 8 |  |
| Female | 3 |  |
| Parasitemia on day 0 (parasites/µl) | 5,170.83 | 200-15,000 |
| Total no. of previous malaria episode (%) | 1 (9.09) | 0 |
